# Increased Activity of Cell Surface Peptidases in HeLa Cells Undergoing UV-Induced Apoptosis Is Not Mediated by Caspase 3

**DOI:** 10.3390/ijms13032650

**Published:** 2012-02-28

**Authors:** Terrence J. Piva, Catherine M. Davern, Paula M. Hall, Clay M. Winterford, Kay A. O. Ellem

**Affiliations:** 1 School of Medical Sciences, RMIT University, Bundoora 3083, Victoria, Australia; 2 QCF Cancer Research Unit, Queensland Institute of Medical Research, Herston, Queensland 4006, Australia; E-Mails: cathyL@qimr.edu.au (C.M.D.); PaulaH@qimr.edu.au (P.M.H.); clayW@qimr.edu.au (C.M.W.); kayE@qimr.edu.au (K.A.O.E.)

**Keywords:** cell surface peptidase, UV radiation, cell death, apoptosis

## Abstract

We have previously shown that in HeLa cells treated with a variety of agents there is an increase in cell surface peptidase (CSP) activity in those cells undergoing apoptosis. The increase in CSP activity observed in UVB-irradiated cells undergoing apoptosis was unaffected when the cultures were treated with the aminopeptidase inhibitor bestatin, and matrix metalloprotease inhibitor BB3103, but greatly enhanced when treated with the caspase 3 inhibitor-DEVD, and reduced in the presence of the poly(ADP-ribose) polymerase (PARP) inhibitor-3-aminobenzamide (3AB). Neither 3AB nor DEVD had an effect on the gross morphology of the apoptotic cells observed under electron microscopy, nor did they have an effect on phosphatidylserine eversion on the cell membrane, or that of PARP cleavage. All the agents except for DEVD had no effect on the level of caspase 3 activity in the cells. The results suggest that other caspases may cleave PARP in these cells. Both 3AB and DEVD treatment reduced the level of actin cleavage seen in the apoptotic cells. The increase in CSP activity observed in cells undergoing UVB-induced apoptosis appears to involve PARP but not caspase 3.

## 1. Introduction

When a cell is exposed to a lethal dose of a genotoxic agent, it can die by one of two known mechanisms, necrosis or apoptosis. These two forms of cell death differ from each other with respect to many aspects of cell biology [1–3]. Since the initial landmark observations made by Kerr and others [4], numerous studies have been undertaken to elucidate the many processes that occur in cells undergoing apoptosis. The gross morphological changes that occur in a cell undergoing apoptosis have been well characterised in the literature. One of the early changes observed in an adherent cell undergoing apoptosis is its detachment from the substrate [5] followed by the loss of specialised membrane structures such as microvilli [6]. The cell then undergoes rounding, shrinkage and blebbing before condensation of chromatin is observed in the nucleus. After a period of time the cell fragments into apoptotic bodies, which *in vivo* are engulfed and degraded by phagocytic cells such as macrophages [7].

Many biochemical changes have been shown to occur in a cell as it undergoes apoptosis. Phosphatidylserine inversion on the cell membrane occurs early during apoptosis while that of carbohydrates such as fucose occurs later [8,9]. Intracellular proteases such as caspases are activated, which result in the cleavage of intracellular proteins such as poly(ADP-ribose) polymerase (PARP), DNA-dependent protein kinase (DNA-PK), actin, fodrin and lamin [10,11]. Other proteases that play a role in apoptosis include calpains and serine proteases [5,12]. Increased metalloprotease activity on the cell membrane has been observed resulting in the shedding of proteins such as CD46, E-cadherin and L-selectin from cells undergoing apoptosis [13–16].

Enzymes on the plasma membrane such as caspase 1 (or ICE), are activated as the cell undergoes apoptosis [10,11]. ICE cleaves IL-1β from its precursor form, which *in vivo* attracts macrophages to engulf these cells as they undergo apoptosis. The mechanisms by which cell surface peptidases (CSP) in HeLa cells [17] are activated during apoptosis still remain to be elucidated. This activation could be a result of phosphatidylserine inversion in the cell membrane [8,9]. However, this does not appear to be the case, as a uniform change in enzyme activity was not observed in these cells as they were exposed to a range of apoptotic stimuli [17]. Changes in CSP activity may be related to intracellular events occurring as the cell undergoes apoptosis. Caspase 3 cleaves a wide range of proteins including PARP, gelsolin and Apaf-1 [10,11], as well as activating caspases 2 and 6 in the cell [18]. The action of this and other caspases may be involved in the activation of these proteases. PARP located in the nucleus is activated in response to metabolic, chemical, or radiation-induced DNA single strand breaks [19,20]. NADH is required by PARP as a substrate for generating ADP-ribose monomers, and excessive DNA damage may deplete the cell of its energy reserves, resulting in necrosis [21]. However, during apoptosis PARP is cleaved by caspase 3, thereby preventing the NADH depletion, and at the same time cell death switches from necrosis to apoptosis [19,20]. It is unknown if PARP cleavage plays a role in activating CSP activity.

In this study, we examined the effect of UVB radiation on HeLa cells, and to elucidate the mechanism(s) involved in activating CSP in cells undergoing apoptosis. It is unknown if the activation of these peptidases [22] is part of a global response to UV radiation or is only observed in cells undergoing apoptosis. CSP activity was increased in UVB-induced apoptotic cells, except in those treated with the PARP inhibitor (3-aminobenzamide, 3AB). In general, UVB-induced necrotic cells had significantly reduced levels of CSP activity, while those seen in unirradiated cultures did not. From the results of studies undertaken it was concluded that CSP activity was modulated by PARP activation but caspase 3 was not involved.

## 2. Results

**Figure 1:**
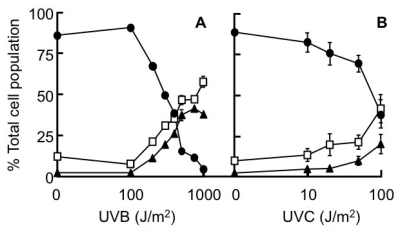
The dose response curves of HeLa cells exposed to UVB- and UVC-radiation. The percentages of the subpopulations [viable (●), apoptotic (▴) and necrotic cells (□)] were calculated where the total population represents 100%. The cells were exposed to either (**A**) UVB or (**B**) UVC radiation. Results expressed are the mean ± SEM of 5–15 samples.

In an earlier study, we have shown that the levels of cell surface peptidase (CSP) activities were increased in apoptotic cells and reduced in necrotic cells compared to that seen in the viable UVC-irradiated HeLa cells [17]. In order to be able to separate and collect viable, apoptotic and necrotic cells using flow cytometry, the HeLa cells were stained with Hoescht 33342 (H33342) and Propidium Iodide (PI) dyes. The cells found in these three regions differed from each other with respect to morphology as well as DNA banding patterns [17]. HeLa cell cultures were exposed to different doses of either UVB or UVC radiation, and were harvested 20 h post-irradiation. From Figure 1, it can be seen that with UVB radiation at <500 Jm^−2^ there were few apoptotic cells, while at >500 Jm^−2^ there were few viable cells. Therefore in order to obtain optimal numbers of all three cell subpopulations, a dose of 500 Jm^−2^ UVB was used. The survival curves of the UVB-irradiated HeLa cells were different to that seen in the same cells exposed to UVC radiation [17]. A similar observation was also observed in HaCaT cells exposed to either UVB or UVC radiation [23].

**Figure 2:**
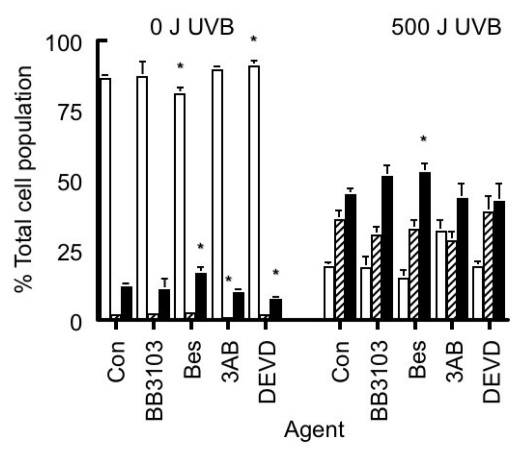
Effect of different agents on the composition of HeLa cell cultures that have been exposed to either 0 or 500 Jm^−2^ UVB radiation. The percentages of the subpopulations were calculated where the total population represented 100%, and are comprised of viable (□), apoptotic (▨) and necrotic cells (■). The following agents were added to the cultures: Nothing (controls), 10 μM Bestatin (Bes), 20 μM BB3103, 10 mM 3AB and 1 μM DEVD. Results expressed are the mean ± SEM of 5–15 samples. The statistical significance of the difference between the control population and the treated groups for each subpopulation are represented as * *p* < 0.05.

In order to elucidate the mechanism involved in activation of CSP in apoptotic HeLa cells [17], we examined the effects of the following agents in UVB-irradiated cells: (a) aminopeptidase inhibitor bestatin (10 μM) [24]; (b) matrix metalloprotease inhibitor BB3103 (20 μM) (kindly supplied from British Biotech Pharmaceuticals); (c) PARP activation inhibitor 3AB (10 mM) [25,26]; and (d) caspase 3 inhibitor DEVD (1 μM) [27]. The effects of these agents on the composition of the different cell subpopulations in sham- and UVB-irradiated HeLa cell cultures are seen in Figure 2. In general, these agents had no effect on the composition of the different cell subpopulations in either irradiated cultures. In the unirradiated cultures, those treated with 10 μM bestatin had less viable and more necrotic cells than untreated control cultures, while those treated with the cell permeable caspase 3 inhibitor, DEVD, had more viable and less necrotic cells. In the UVB-irradiated cultures bestatin treatment increased the number of necrotic cells compared to that seen in untreated irradiated cultures.

### 2.1. Cellular Protein Content

The protein content of the apoptotic and necrotic subpopulations of the sorted UVB-irradiated cells were less than that of their corresponding viable cohorts (Table 1). The results obtained were similar to that seen when HeLa cells were exposed to different apoptotic stimuli [17]. The necrotic cells contained approximately one-third of the protein content of their viable cell cohorts in the unirradiated cultures. The protein content of the apoptotic cell subpopulation of the unirradiated cultures could not be determined due to the low number of cells present. The protein content of the viable subpopulation of the UVB-irradiated cultures were approximately 80% of that of the corresponding unirradiated cultures except for those cells treated with DEVD (58%). The protein content of the apoptotic subpopulation of the UVB-irradiated cultures apart from the controls (83%), was similar to that of their corresponding viable cohorts (92–100%). The necrotic cell fraction contained 40–52% of the protein content of their viable cohorts. These changes in the protein content of apoptotic and necrotic cells were similar to that seen previously [7,17,28]. This reduction in protein content is most likely due to a disruption of plasma membrane integrity resulting in the loss of cytosolic proteins [7,28].

### 2.2. Cell Surface Peptidase (CSP) Activity

In measuring the level of CSP activity in these cells, we used a nonapeptide substrate (called P9) that is cognate to the *N*-terminal cleavage site of TGFα (transforming growth factor-α) [17,22,29]. The activity of the “TGFαase”, which cleaves the P9 peptide at the *N*-cleavage site corresponding to that seen in TGFα, produces a P5 fragment, has been shown to be elevated in UV-irradiated cultures [17,22,30]. It is generally acknowledged that TACE (tumour necrosis factor α converting enzyme) is responsible for the cleavage of TGFα at both its *N*- and *C*-terminal cleavage site [31,32], though it has been suggested that other enzymes may be involved [33]. In this study we refer to the enzyme responsible for the production of the P5 fragment from P9 as TACE.

**Figure 3:**
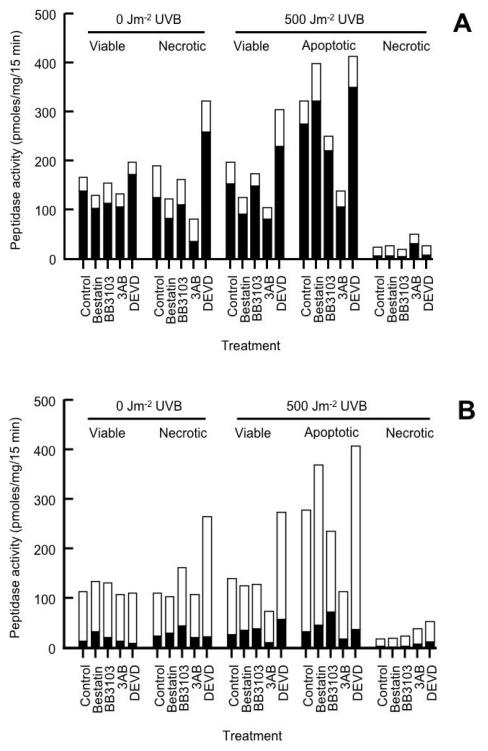
Comparison of the level of cell surface peptidases (CSP) activity in the cell subpopulations of HeLa cells treated with different agents. The level of aminopeptidase (□) and ectopeptidase (■) activity was shown in the cell subpopulations of sham- or 500 Jm^−2^ UVB-irradiated cells in the absence (**A**) or presence of 10 μM bestatin (**B**).

The level of CSP activity in the different cell subpopulations can be seen in Tables 2 (sham-irradiated cells) and 3 (UVB-irradiated cells). Total CSP activity was similar between the viable and necrotic subpopulation of unirradiated cells (Figure 3). The main peptidase present on the HeLa cell membrane was an aminopeptidase [17,22,30]. In HeLa cells this is not aminopeptidase N [34]. This aminopeptidase accounts for between 79–86% total CSP activity in the viable subpopulation of the unirradiated cultures, and 64–77% in the necrotic subpopulations (Table 2). CSP activity was not measured in the apoptotic subpopulation of the unirradiated cultures, as there were insufficient numbers of cells to perform these assays. Of the treatments used in the unirradiated cultures, DEVD induced aminopeptidase activity in both the viable and necrotic cell subpopulations, while 3AB inhibited activity in the necrotic cells (Table 2).

The aminopeptidase inhibitor bestatin [24,35] significantly reduced aminopeptidase activity in the cells (Tables 2 and 3). As a result, the levels of dipeptidase, tripeptidase and TACE activity were increased, with the tripeptidase having the highest activity. We will use the term ectopeptidases to refer to dipeptidase, tripeptidase and TACE activity, as these enzymes act at cleavage sites within the P9 substrate. In general, in the presence of bestatin, the level of CSP activity was similar in the viable (108.3–134.8 pmol/mg/15 min) and necrotic cell subpopulations (103.9–265.6 pmol/mg/15 min) of the unirradiated cells. A significant increase in tripeptidase activity (316%) was observed in the necrotic cell subpopulation of DEVD-treated cells compared to that seen in untreated cultures (Figure 3B).

Previously we observed that CSP activity (aminopeptidase and TACE) was increased 2-fold in UVC-irradiated cells [22]. Like that seen in the unirradiated cultures, the main CSP present in the UVB-irradiated cells was an aminopeptidase (Table 3). With the exception of the 3AB-treated cells (87%), the level of aminopeptidase and that of total CSP activity present in the viable subpopulations of UVB-irradiated cultures were similar to that seen in the viable subpopulations of unirradiated cultures (Figure 3A). DEVD increased both aminopeptidase and total CSP activity in the viable subpopulation of the UVB-irradiated cells when compared to their untreated cohorts.

Both aminopeptidase and total CSP activity was significantly increased in the apoptotic fraction of all the treated UVB-irradiated cells compared to their corresponding viable cell cohorts except for those cultures treated with 3AB (Figure 3A). The greatest increase in aminopeptidase and total CSP activity between the apoptotic and viable cells of the UVB-irradiated cultures was observed following bestatin treatment (240 and 222%, respectively). In neutrophils increased expression of aminopeptidase N was seen on the plasma membrane in cells undergoing apoptosis [36].

In the UVB-irradiated necrotic cells both aminopeptidase and total CSP activity was significantly lower in the necrotic subpopulations when compared to their respective viable or apoptotic cohorts (Figure 3A). This result suggests that these CSP have been shed from the cell membrane, which was similar to that seen in the necrotic subpopulations of UVC-radiated cells [17].

In general, in the UVB-irradiated cultures, the level of ectopeptidase activity was similar in the viable subpopulation of the treated cells compared to the same cohort in the unirradiated cultures (Figure 3B). Elevated levels of ectopeptidase activity were seen in the viable subpopulation of the DEVD-treated (195%) but lower in the 3AB-treated (53%) UVB-irradiated cells compared to their corresponding viable cohorts. In those cells undergoing apoptosis, there was a significant increase in ectopeptidase activity (150–294%) compared to that seen in the viable subpopulation irrespective of the treatment (Figure 3B). The greatest increase in ectopeptidase activity was seen in bestatin-treated cells (294%) with the least seen in DEVD-treated cells (150%). Most of this increase was due to elevated tripeptidase activity (130–298%). While dipeptidase activity was less than that of tripeptidase, its activity also increased in the apoptotic subpopulations (138–383%) of the treated cells compared to their corresponding cohorts of viable cells. TACE activity was higher in the apoptotic subpopulation of BB3103-treated cells (217%) otherwise its levels of activity were similar to that of its corresponding viable cohorts. In the necrotic cells, irrespective of the treatments used, the level of ectopeptidase activity was significantly reduced compared to that seen in the viable or apoptotic cell subpopulations (Figure 3B). The results obtained were similar to that seen in an earlier study where increased CSP activity was observed in cells undergoing apoptosis while there was a loss in activity in the necrotic cells [17]. It is not clear as to how DEVD increases or 3AB decreases CSP activity in the viable and apoptotic subpopulations of UVB-irradiated cells or that of the necrotic subpopulation of unirradiated cultures.

### 2.3. Cell Morphology

**Figure 4:**
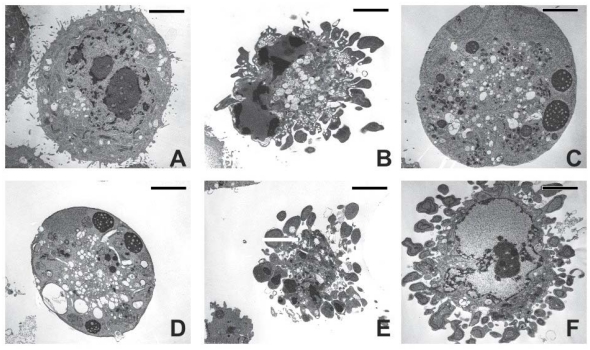
Electron micrograph of apoptotic HeLa cells from cultures treated with different agents. The cells were treated with the following agents: (**A**) Sham-irradiated viable cells; (**B**) UVB-irradiated (500 Jm^−2^) cells; (**C**) Bes; (**D**) BB3103; (**E**) 3AB; and (**F**) DEVD. Bar = 5 μm.

In order to elucidate the effect 3AB and DEVD treatments had on CSP activity in the apoptotic cells, we looked at their morphology. The effects of the treatments on the morphology of the apoptotic cells are seen in Figure 4. Both 3AB and DEVD treatments had no effect on the morphology of UV-induced apoptotic cells, as evidenced by the formation of apoptotic bodies and condensation of the chromatin to the nuclear membrane [1,3,4]. Of interest was the morphology of Bestatin or BB3103-treated apoptotic cells (Figure 4C,D). These cells appear to have morphology similar to that of necrotic cells, as no apoptotic bodies appear to be present. There were increased levels of lipid droplets present in these cells, and the mitochondria appear extended, however, this morphology was similar to that seen in apoptotic human A549 lung adenocarcinoma cells treated with antisense matrilysin oligonucleotides [37]. The effects induced by either 3AB or DEVD on CSP activity in the apoptotic cells do not appear to be related to changes in cell morphology.

### 2.4. Annexin V-Staining Profiles

**Figure 5:**
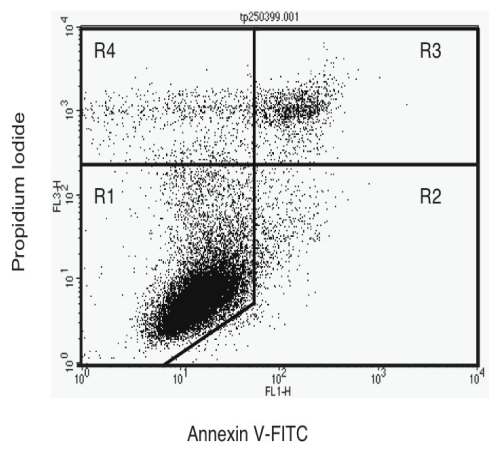
Separation of HeLa cell subpopulations by flow cytometry. The different subpopulations of cells are R_1_ = Viable cells, R_2_ = Early Apoptotic cells, R_3_ = Late Apoptotic/Necrotic cells and R_4_ = Cell debris.

As the morphology of the 3AB or DEVD-treated UVB-induced apoptotic cells did not differ to that of untreated apoptotic cells, we then investigated if the changes in CSP activity was related to phosphatidylserine levels on the outside of the cell membrane. Through using FITC-labelled annexin V and PI staining, it is possible to distinguish different cell subpopulations as seen in Figure 5. The intensity of annexin V-staining of the apoptotic cells was approximately one order of magnitude higher than that of their viable cell cohorts, in agreement to that seen previously [38].

It was not until 12 h post-UVB radiation that there was a significant increase in the level of annexin V-staining occurring on the surface of HeLa cells (results not shown). There was a sharp increase in the number of late apoptotic/necrotic cells occurring in the irradiated cells after 12 h, which was accompanied by a corresponding drop in the number of viable cells, while the numbers of early apoptotic and damaged cells did not change. We also observed that the viability rapidly dropped in cultures exposed to >100 Jm^−2^ UVB 20 h post-irradiation (Results not shown), which was similar to that seen when the cells were stained with PI and H33342 (Figure 1).

**Figure 6:**
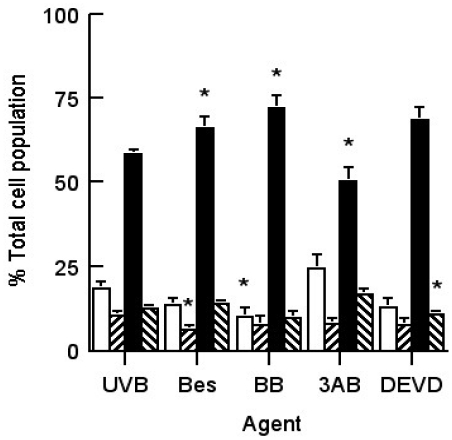
Effect of different agents on annexin V-staining of UVB-irradiated HeLa cell cultures. UVB-Irradiated (500 Jm^−2^) cells were exposed to different agents (UVB radiation (UVB), Bestatin (Bes), BB3103 (BB), 3-aminobenzamide (3AB) and DEVD). The different cell subpopulations were: Viable (□), Early apoptotic (▧), late apoptotic/necrotic (■) and Cell debris (▧). Results expressed are the mean ± SEM of 3 separate experiments. The statistical significance of the difference between the % total cell subpopulation between the untreated controls and each treatment group is represented as * *p* < 0.05.

The effect of the different treatments on phosphatidylserine eversion on the cell membrane in 20 h post UVB-irradiated cultures is seen in Figure 6. Cells treated with bestatin, BB3103 and DEVD had similar annexin V/PI staining profiles compared to that seen in the untreated irradiated cultures. There was an elevated number of viable cells and a corresponding reduced number of late apoptotic/necrotic cells in the 3AB-treated cultures. However, these differences were not statistically different and did not correspond to the changes in the level of CSP activity observed in either the 3AB- or DEVD-treated cultures.

### 2.5. PARP Cleavage

**Figure 7:**
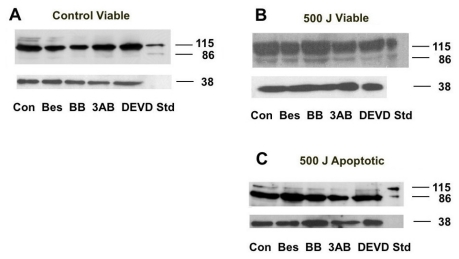
Effect of different agents on PARP cleavage in different fractions of irradiated HeLa cell cultures. UVB-irradiated cells (0 Jm^−2^ or 500 Jm^−2^) were treated with the following agents: nothing (controls: Con), bestatin (Bes), BB3103 (BB), 3AB and DEVD. Lysates of sorted viable subpopulations of unirradiated cultures (**A**); and UVB-irradiated cultures (**B**) along with the apoptotic subpopulations of UVB-irradiated cultures (**C**) were prepared as described in the Materials and Methods section. Jurkat cell lysates (Std) were added to each gel. In the 500 J Viable blot 40 μg cell lysate was added per lane while in the other blots 20 μg was added. GAPDH was used as a loading control. Molecular mass markers in kD are indicated on the right.

3AB is known to inhibit PARP activation in cells suffering oxidative stress [20,39]. We therefore decided to see if 3AB affected the cleavage of PARP by the caspases, which could explain the differences in CSP activity observed in the treated cultures. Cell lysates from the viable, apoptotic and necrotic subpopulations of UVB-irradiated cells treated as well those from the viable subpopulation of unirradiated cells treated with different agents were prepared from Flow cytometry-sorted cells. A Western blot of the cell lysates (20 μg cell protein) is seen in Figure 7. In the viable fraction of the unirradiated cells PARP remained intact 20 h-post treatment, irrespective of treatment. Similar results were also seen in the viable subpopulations of the UVB-irradiated cells. However, in both the apoptotic and necrotic fractions of these treated cell populations PARP (115 kD) was cleaved to its 86 kD product. The caspase 3 inhibitor DEVD did not prevent PARP from being cleaved, which suggests that other caspases such as caspase 7 may be present [11,21]. The addition of 3AB to the UVB-irradiated cells did not prevent PARP cleavage in the apoptotic cells, which suggests that the activation of CSP activity is not mediated by caspase 3.

### 2.6. Caspase 3 Activity

**Figure 8:**
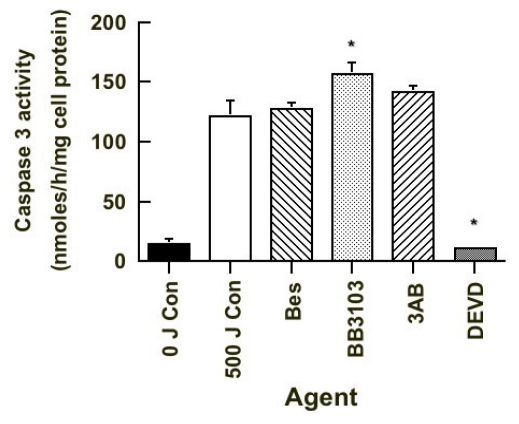
Effect of different agents on caspase 3 activity in irradiated HeLa cell cultures. The level of caspase 3 activity was measured in sham-irradiated untreated cultures (0 J Con) (■), and in UVB-irradiated cultures treated with nothing (500 J Con) (□), 10 μM Bes (▧), 20 μM BB3103 ( 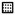), 10 mM 3AB (▨) and 1 μM DEVD ( 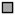). Results expressed are the mean ± SEM of 3 separate experiments. The statistical significance of the difference between caspase 3 activity in the control population and in each treated group is represented as * *p* < 0.05.

As PARP was cleaved in apoptotic cells treated with DEVD, we then measured the level of caspase 3 activity in the 20 h post-UVB irradiated cultures, in order to confirm if other caspases are present. Initial studies using flow cytometry-sorted cells were unsuccessful as it was not possible to obtain sufficient numbers of cells in a short period of time to be able perform this assay. We used cells obtained from UVB-irradiated cultures treated with different agents, as this would give an indication of the level of caspase 3 activity present. The results of which are seen in Figure 8. As expected the level of caspase 3 activity was low in the untreated sham-irradiated cultures and much higher in untreated UVB-irradiated cells. Neither bestatin or 3AB treatment had an effect on caspase 3 activity, while BB3103 significantly increased and DEVD significantly inhibited activity. The DEVD result was expected as it is a caspase 3 inhibitor [10,11]. The results suggest that other caspases (e.g. caspase 7) may be responsible for the cleavage of PARP in these cells (Figure 7). It also suggests that caspase 3 does not directly modulate CSP activity in cells undergoing UVB-induced apoptosis.

### 2.7. Actin Cleavage

**Figure 9:**
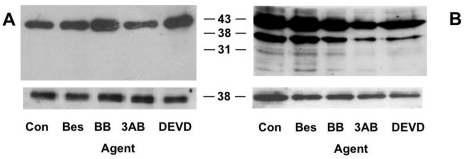
Effect of different agents on the cleavage of actin in the apoptotic population of irradiated HeLa cell cultures. Lysates from the sorted apoptotic subpopulations of treated (controls (Con), bestatin (Bes), BB3103 (BB), 3AB and DEVD) (**A**) or the viable subpopulation (Via) of untreated 500 Jm^−2^ UVB-irradiated cultures (**B**) were run on Western blots. GAPDH was used as a loading control. Molecular mass markers in kD are indicated on the right.

It has been shown that 3AB prevents actin cleavage, which confers a protective effect to cells preventing them from undergoing apoptosis [10,25]. A Western blot of cell lysates from the apoptotic subpopulation of 20 h post-UVB irradiated cell cultures was probed with an actin antibody as seen in Figure 9. In the apoptotic subpopulation of the treated UVB-irradiated cells the 43 kD actin protein was cleaved to produce 38 and 31 kD actin fragments, but the 14 kD fragment was not observed in cultures treated with either 3AB or DEVD. Actin degradation was not seen in the viable untreated irradiated cells. These results suggest that the changes in CSP activity seen in the cells as they undergo apoptosis is not due to actin cleavage, because 3AB-treatment reduced CSP activity, while that of DEVD enhanced activity, but both elicited the same inhibitory effect on actin cleavage. This suggests the mechanism involved in modulating CSP activity in these cells undergoing UVB-induced apoptosis does not involve caspase 3, PARP or actin cleavage, and occurs by another mechanism. Further studies on which are currently underway.

## 3. Discussion

Ultraviolet (UV) light is a form of electromagnetic radiation that is emitted by the sun. It can be subdivided into three components, based on wavelength: UVC (200–280 nm), UVB (280–320 nm) and UVA (320–400 nm). Most of the UV radiation that reaches the Earth’s surface consists of UVA (320–400 nm) (90–95%) along with a smaller amount of UVB (280–320 nm) (5–10%). While only a small amount of UVB reaches the Earth’s surface, it is of significance as it is 1000 times more erythrematogenic than UVA [40]. UV radiation that is absorbed by epidermal cells (e.g., keratinocytes and melanocytes) triggers a number of signalling pathways, which in turn elicits different cellular responses [40,41]. Sunburn is caused by keratinocytes undergoing apoptosis as a result of high levels of UV exposure [1,2,23,39,40]. While many intracellular processes are altered as a result of these cells undergoing apoptosis, changes that occur on the cell membrane with regard to enzyme activity are not well characterized.

Even though HeLa cells are not an epidermal cell, they are epithelial in origin, and like HaCaT cells (an immortalized keratinocyte cell line), they possess a dysfunctional p53 [42,43]. In human skin p53 mutations are commonly observed in the sun-exposed noncancerous epidermis, and it is from these mutated cells that squamous cell carcinomas (SCC) have been shown to develop [44]. In keratinocytes that have a functional p53, excessive exposure to UV radiation results in these cells undergoing apoptosis [1,2,45]. As a result of UV-induced DNA damage p53 is activated, which in turn upregulates p21 levels and through downstream signalling the cell will undergo apoptosis if this damage cannot be repaired [45]. If p53 is mutated, p21 activation is much weaker, and as a result fewer irradiated cells undergo apoptosis, and those surviving cells have the potential to become cancerous. Therefore in order to observe the effect of UVB radiation in squamous cell carcinoma development, keratinocytes that harbour p53 mutations such as HaCaT cells are a good experimental model [44,45]. As the numbers of cells needed to undertake a study of CSP activity are high, and their separation requires flow cytometry to collect cells from viable, apoptotic and necrotic subpopulations, we used HeLa cells in this study. These cells can easily be grown in large numbers and like HaCaT cells they possess a dysfunctional p53 [42,43]. The results obtained from this study can be used to devise further experiments using HaCaT cells which will allow us to understand the role played by mutated keratinocytes (possessing a dysfunctional p53) found in actinic keratosis [46] following their exposure to UV radiation.

We had shown previously that cycloheximide did not inhibit enhanced CSP activity in apoptotic cells [17], which suggested that increased activity was not due to *de novo* synthesis and this activation was by another mechanism. Therefore, in order to further our understanding of this mechanism, we investigated the effect different inhibitors had on UVB-induced apoptotic cells. Bestatin inhibits a range of aminopeptidases as well as Leukotriene A4 hydrolyase [24,35]. The cells were incubated with bestatin in order to observe if aminopeptidases activate CSP as a result of the cells undergoing apoptosis. BB3103 is a MMP inhibitor and was added to the cells to observe if these membrane proteins activate CSP activity. 3AB prevents the activation of PARP, and it was used to observe if PARP activation played a role in modulating CSP activity. DEVD is a caspase 3 inhibitor as was used to see if caspase activity modulated CSP activity, we have previously shown that ICE (caspase 1) found on the cell membrane had no effect on activity [17].

The viability profiles of HeLa cells exposed to UVB and UVC radiation differed (Figure 1) and were probably a result of different apoptotic pathways being activated in the cell. UVC radiation generated more (6–4) photoproducts and cyclobutane-pyrimidine dimers than did UVB in HaCaT cells [23] and it is likely that this occurred in the irradiated HeLa cells. It has been shown in keratinocytes and HaCaT cells, UVB radiation can initiate apoptosis by three major mechanisms (a) DNA damage, (b) reactive oxygen species (ROS) and (c) the activation of death receptors [1,2,23,44]. UVC mainly exerts its apoptotic effect by initiating DNA damage. The pathway by which UVC induces apoptosis is called the intrinsic pathway and it differs from the extrinsic pathway induced by UVB, because it does not involve caspase 8 and related caspases which is stimulated as a result of the activation of death receptors (e.g., Fas) on the cell membrane [23,47]. A unique feature of the extrinsic pathway is that it can trigger apoptosis independently of p53 [48]. Therefore it is likely that the differences seen in HeLa cells undergoing apoptosis as a result of UVC [17] and UVB radiation (Figure 1) are due to the different apoptotic pathways (intrinsic and extrinsic) that are activated by these forms of UV radiation.

The main peptidase found on the plasma membrane of the HeLa cells was an aminopeptidase (Figure 3). In the unirradiated cultures the level of CSP activity was similar in the viable and necrotic subpopulation of cells. This suggests that there has been neither an upregulation of CSP activity nor a shedding of these peptidases from the cell membrane as a result of the cell dying. If aminopeptidase activity was inhibited by the addition of bestatin, a specific aminopeptidase inhibitor, the overall level of CSP was similar which suggests that the ectopeptidase products (P2, P3 and P5) are further degraded by the action of the aminopeptidase. Aminopeptidases found on the cell membrane process a range of substrates, and play a role in many biological processes, as well as disease states [35,49]. They can also cleave the *N*-terminal amino acid from peptides or proteins, which can then in turn be taken up by the cell via specific transporters as part of a salvage type mechanism [30,35,50]. The actions of which can also allow the cell to obtain essential amino acids from the cleavage of peptides and proteins on the plasma membrane.

In the UVB-irradiated cells enhanced CSP activity was not seen in the viable cell subpopulation, unlike that seen in the apoptotic subpopulation where it was increased, and reduced in the necrotic subpopulation (Figure 3). This finding suggests that UVB-radiation does not have an effect on CSP activity as no increase in activity was observed in the viable cells, and the increased activity seen in apoptotic cells arises when these cells undergoing apoptosis. Of interest was that 3AB treatment reduced CSP activity, while that of DEVD enhanced activity in both the viable and apoptotic subpopulations of the UVB-irradiated cells. Bestatin only enhanced CSP activity in cells undergoing apoptosis, while BB3103 had no effect. This increase in CSP activity in the apoptotic subpopulation compared to the viable subpopulation is not related to changes in cell protein levels (Table 1) as if this was the case enzyme activity would only be increased 1.25-fold. Increased CSP activity as high of 2.2-fold was observed in the apoptotic subpopulations compared to the viable subpopulations of the irradiated cells. As the increase in CSP activity was not uniform across all treatments, this suggests that it is not part of a global apoptotic response. In a previous study we observed increased CSP activity in cycloheximide-induced apoptotic cells, which suggests that this increased activity is not due to the synthesis of new proteins in these cells [17]. Increased CSP activity may arise from an activation of existing enzymes on the cell membrane or as a result of increased translocation of enzymes from the cytoplasm to the plasma membrane, in an analogous manner to how insulin stimulates the relocation of GLUT-4 transporters from the cytoplasm to the plasma membrane [51].

The reduction in CSP activity in the necrotic cell subpopulation (Figure 3), suggests that these enzymes are shed from the cell membrane as the cells undergo necrosis. It has been shown previously that UVC-induced necrosis resulted in a loss of cellular protein and a corresponding loss of CSP activity [17]. It has been shown that a range of pro-inflammatory molecules (e.g., IL-6, uric acid) are shed from the surface of necrotic cells [52]. It is not known if proteases released from the surface of necrotic cells directly contribute to the release of pro-inflammatory molecules or may contribute to the dissolution of matrix components surrounding these cells.

The increase in CSP activity observed in cells undergoing apoptosis most likely has a specific physiological function. Some of the membrane proteins shed from apoptotic cells include cytokines (IL-1β and TNFα), adhesion molecules (e.g., E-cadherin and L-selectin), receptors (CD15, CD16, IL6R, TNF-R1 and TNF-R2) as well as other proteins (e.g., CD46) [13–15,34,36,53]. Some of these molecules are known to attract phagocytic cells to ensure the removal of the apoptotic bodies, as well as downregulating an inflammatory response. While these proteins are shed during the apoptotic process, it is unknown whether the activities of the enzymes involved are either (a) upregulated or (b) there are increased numbers present on the cell membrane. Apart from that of caspase 1 [10,11], both aminopeptidase N [36] and TACE activity [15] have been shown to be increased in apoptotic neutrophils, along with that of CSP in UVC-induced apoptotic HeLa cells [17]. As seen in this study the level of enzyme activity increased from 1.5–2.2-fold for each of the peptidases that were investigated. This increased level of CSP activity may result in the release of bioactive molecules which can (a) assist in attracting phagocytic cells to remove the resultant apoptotic cells, (b) reducing inflammation, as well as (c) stimulating the growth of surrounding unaffected cells to replace those which have died. The latter could be one mechanism by which surviving epidermal keratinocytes are stimulated to grow and replace those sunburnt cells lost as a result of UV exposure [39,40,54]. Cell surface proteases have been shown to play an important role in wound repair and tissue growth [55], and one can speculate that following the loss of sunburnt cells, that these proteases as a result of being activated would be involved in stimulating tissue growth in these cells, and this process would most likely commence when these cells undergo apoptosis.

The caspase 3 inhibitor Ac-DEVD-cho enhanced CSP activity in the necrotic subpopulation of unirradiated cultures as well as that of the viable and apoptotic subpopulations of UVB-irradiated cells (Figure 3). Caspase 3 is an executioner caspase and is known to cleave over 200 cellular proteins [10,11,56]. It is estimated through the use of bioinformatics that there are at least 3000 human proteins that contain a caspase 3 cleavage site [56]. The inhibitor Ac-DEVD-cho was shown to inhibit caspase 3 activity (Figure 8) and therefore one can speculate that this enzyme may cleave protein(s) that regulate the activation of the CSP observed in this study. As both the aminopeptidase and ectopeptidases were activated in the necrotic subpopulation of the unirradiated cultures as well as that of the viable and apoptotic subpopulations of the UVB-irradiated, it is unlikely that this activation is related to the activation of apoptotic pathways, but occurs via another unknown mechanism. Pan caspase inhibitors such as Z-VAD-FMK and Q-VD-OPH, when added in combination have been shown to inhibit most of the caspases in the cell [57]. The results of such a study would confirm if the activation of CSP seen in the apoptotic cells is related to that of the caspases.

The PARP activation inhibitor 3AB [19–21] elicited an opposite effect to that of DEVD with regards to CSP activity in these cells (Figure 3). 3AB significantly reduced CSP activity in the necrotic subpopulation of unirradiated cultures as well as in the viable and apoptotic subpopulation of UVB-irradiated cultures. When PARP becomes activated it is involved in repairing damaged DNA, and requires NADH to be able to effect this repair [19–21]. When the cell suffers significant DNA damage, in this case UVB radiation, in order to prevent the depletion of NADH stores which will result in the cell undergoing necrosis, and resultant inflammation, the cell initiates the caspase cascade. This result in the cleavage of PARP, preventing the depletion of cellular NADH stores, resulting in the cell undergoing apoptosis, and reducing the inflammatory response. PARP inhibitors at high concentrations (10 mM) have been shown to inhibit matrix metalloprotease 2 (MMP-2) activity on the cell membrane [58]. MMP-2 is a metalloprotease, and the enzyme furin is responsible for its activation [59], one can speculate that 3AB inhibits the activity of this proprotein convertase, resulting in the reduced expression of this metalloprotease on the cell membrane. In the current study it is possible that 3AB inhibits either the activation or expression of CSP on the surface of these cells. In order to understand how 3AB is exerting its effects further studies are warranted. A measure of the cellular NADH levels as well as that of PARP activity may shed some light on whether CSP activation is energy dependent, this may elucidate why 3AB and DEVD treatment results in differing levels of CSP activity in the cell. The use of other structurally distinct PARP inhibitors (e.g., PJ34, 1,5-isoquinolinediol) may also help elucidate the role PARP plays in modulating CSP activity.

When investigating the mechanism by which DEVD or 3AB affect CSP activity, we observed that changes in the level of phosphatidylserine eversion on the cell membrane (Figure 6) did not correlate to the observed changes in CSP activity. The gross morphology of the apoptotic cells treated with DEVD and 3AB was also similar to that seen for untreated apoptotic cells (Figure 4). These findings suggest that changes in CSP activity are not due to changes in cellular morphology or that of lipids on the cell membrane. The morphology of the apoptotic cells obtained from cultures treated with bestatin and the metalloprotease inhibitor BB3103 does not appear to contain apoptotic bodies. It has been shown that MMP-7 inhibition resulted in altered morphology of apoptotic cells where the mitochondria were swollen and the endoplasmic reticulum was dilated [37]. Recently it has been shown that in some cells swelling precedes cell shrinkage and the formation of apoptotic bodies [60]. The bestatin and BB3103-treated apoptotic cells in this study display a similar morphology as described above, and the mechanism involved in the apoptotic pathway of these cells where enzyme inhibitors have been used could be responsible for these morphological changes.

In all the apoptotic treated cells PARP was shown to be cleaved (Figure 7), and while the caspase 3 inhibitor, DEVD, inhibited activity (Figure 8), it suggests that other caspases are involved in this process. Caspase 7 can also cleave PARP [10,11], and is known to be activated in UV-irradiated HeLa cells [61]. Therefore the activation of caspase 7 as a result of UV radiation can overcome the inhibition of caspase 3 and result in the cleavage of PARP as seen in the DEVD-treated cells. Changes in caspase 3 activity (Figure 8) did not correlate to the changes seen in the level of CSP activity in these cells (Figure 3). This suggests that this caspase does not play a direct role in modulating CSP activity. Of interest is that the level of actin cleavage in the treated apoptotic cells was less in the 3AB- and DEVD-treated cells compared to that seen in other treated cells. Caspase 3 is known to cleave actin to smaller fragments and 3AB can prevent cleavage [25]. DEVD inhibits the action of caspase 3, and it suggests that it plays a role in cleaving this cytoskeletal protein [10,11]. This finding supports the proposal that caspase 3 is not involved in the changes of CSP activity in those cells undergoing apoptosis. However the mechanism involved does warrant further investigation.

The results of this study show that in general, when UV-irradiated cells undergo apoptosis, that there is an increase in CSP activity and it is not related to caspase 3 activity. While HeLa cells are epithelial cells they are not epidermal in origin like keratinocytes. It has been shown that primary keratinocytes have a functional p53, while HaCaT cells [1,2,44,45] and HeLa cells [42] do not. The resultant DNA damage induced by UV radiation on keratinocytes results in the activation of p53, which in turn induced p21 expression in the cell, which prevents these cells from entering the S phase of the cell cycle [45]. If the level of damage is too great to repair, p53 will induce the cell to undergo apoptosis [44]. This mechanism removes those cells with damaged DNA thereby preventing them from passing on these defects to daughter cells, which could result in the formation of SCC [44]. As a result of prolonged sun exposure actinic keratoses are formed in the epidermis which contain mutated p53 [46], which can give rise to SCC [44]. HaCaT cells are similar to the actinic keratoses in that they also have a dysfunctional p53, and that they would be a good model in which to investigate the carcinogenic effects of UV radiation. From the results obtained in this study one can speculate that when actinic keratoses undergo UV-induced apoptosis, they may release a range of bioactive molecules, which stimulate the growth of the surrounding surviving mutated cells. If these dividing cells pass on UV-induced DNA mutations, this process if repeated a number of times may result in the development of a SCC. Further investigation on the effect UV radiation on CSP activity in both UV-irradiated HaCaT cells and keratinocytes may yield information on the biological role cell surface proteases play in this process. As a result of the development of pharmacological agents that prevent the activation of these CSP, these agents may play an important preventative role in the formation of skin cancer.

## 4. Experimental Section

### 4.1. Materials

All chemicals and biochemicals were obtained from sources previously described [17,22] except for caspase 3 substrate *N*-acetyl-Tyr-Val-Ala-Asp-p-nitroanilide (Ac-DEVD-pNA), 3-Aminobenzamide (3AB) and the cell permeable inhibitor *N*-acetyl-Tyr-Val-Ala-Asp-CHO (DEVD) which were obtained from Biomol (Plymouth Meeting, PA, USA), rabbit anti-actin polyclonal antibody was obtained from Sigma (St. Louis, MO, USA) rabbit anti-GAPDH (glyceraldehyde-3 phosphate dehydrogenase) monoclonal antibody was obtained from Cell Signaling (Danvers, MA, USA), Jurkat apoptotic lysate kit, fluorescein isothiocyanate-labelled Annexin V and mouse anti-human PARP monoclonal antibody were obtained from PharMingen (San Diego, CA, USA), anti-mouse Ig-peroxidase conjugate was obtained from Transduction Laboratories (Lexington, KY, USA), anti-rabbit Ig-peroxidase conjugate antibody was obtained from Silenus (Melbourne, Australia) and ECL was obtained from NEN (Boston, MA, USA).

### 4.2. HeLa Cell Culture

HeLa S3 cell monolayers were grown in RPMI 1640 containing penicillin (100 μg/mL), streptomycin (100 μg/mL), Hepes (20 mM) and 5% (v/v) foetal bovine serum (FBS) (pH 7.2 at 37 °C) under sterile conditions. Trypsinised cells from 3–4 confluent 175 cm^2^ tissue culture flasks were collected and washed with Phenol red free-HBSS prior to being suspended at 10^7^ cells/mL HBSS. One ml aliquots of cells (10^7^) were transferred to 60 mm tissue culture dishes (Iwaki Glass, Chiba, Japan) which were subsequently irradiated with either 0 or 500 J UVB (FS-20 fluorescent tube generating a flux of 2.1 Jm^−2^/s). The cells were then transferred to a 175 cm^2^ flask containing 50 mL RPMI plus 2% FBS as well as one of the following inhibitors: no inhibitor (control cells), Bestatin (10 μM), BB3103 (20 μM), DEVD (1 μM) or 3AB (10 mM). In the caspase 3 inhibition studies, the cells were pre-exposed to DEVD for 60 min prior to irradiation to ensure that caspase 3 activity was minimal [27]. The cells were placed in a 37 °C incubator for 20 h before being harvested for FACS analysis.

### 4.3. Preparation of Cells for Sorting by Flow Cytometry

The detached cells present in tissue culture media of the treated cultures were transferred to 50 mL Falcon tubes. The attached cells were removed by trypsinization and placed in the same tubes and the samples treated as described previously [17]. Briefly, following centrifugation (37 °C at 200 g for 5 min) the cells were resuspended at 37 °C in 5 mL RPMI containing 2% FBS. The cell populations were then stained with H33342 (1 μg/mL final concentration) for 15 min before being placed on ice for 5 min before being centrifuged (4 °C at 200 *g* for 5 min). The cells were resuspended at 4 °C in 1 mL RPMI containing 2% FBS to which PI (5 μg/mL final concentration) was added. The cells were passed through gauze into 5 mL Falcon tubes prior to being sorted on a Becton Dickinson FACS Vantage. The number of cells found in each fraction was determined using the Cell Lysis analysis software.

### 4.4. Flow Cytometry

The stained cells were separated into viable, apoptotic and necrotic populations on the basis of their staining for PI and H33342 dyes as described previously [17]. The characteristic of staining patterns for the different cell subpopulations were as follows: viable cells possess low H33342 and PI staining, apoptotic cells had a high H33342 and low PI staining, and necrotic cells have high H33342 and PI staining (see Figure 1 [17]). The membrane integrity of early apoptotic cells prevent the entry of PI, and while H33342 binds to the DNA during this period it allows for the cell subpopulations to be distinguished [28,34,62]. The sorted cell subpopulations of UVB-irradiated cells displayed the following characteristics: viable cells possessed intact DNA, apoptotic cells laddered DNA and necrotic cells smeared DNA (Results not shown), which was similar to that seen previously [17]. The morphological characteristic of these irradiated subpopulations were as follows: viable cells (>95%) has an intact plasma membrane and no nuclear chromatin condensation, apoptotic cells (90–95%) possessed apoptotic bodies and condensed chromatin on the plasma membrane, while the necrotic cells (>95%) possessed disrupted plasma membrane and little visible chromatin (Results not shown), which was similar to that seen previously [17]. The sorted cell populations were centrifuged (200 *g* for 5 min at 4 °C) before being washed twice with ice cold HBSS. The sorted cell subpopulations were washed to remove any serum present in the collection medium, as FBS displays some protease activity [22]. After the final wash, the cells were suspended at 10^6^ cells/mL HBSS and 50 μL aliquots placed in microfuge tubes. These tubes were placed in a 37 °C incubator for 15 min to warm the cells prior to being assayed for CSP activity.

### 4.5. Electron Microscopy

The sorted cell populations were centrifuged (200 g for 5 min at 4 °C) before being washed with ice-cold HBSS. The cell pellets were fixed in 3% glutaraldehyde in 0.1 M sodium cacodylate buffer, pH 7.2 for 2 h. The samples were then post-fixed in 1% osmium tetroxide, stained “en block” with 5% uranyl acetate then embedded in epoxy resin. Sections were taken and observed in a Joel 1200 EXII electron microscope.

### 4.6. Annexin V Staining

HeLa cell cultures were grown to near confluency in 60 mm petri dishes (Iwaki Glass, Chiba, Japan) prior to being exposed to different doses of UVB-irradiation as described above. The cells were then given fresh media (RPMI plus 5% FBS) and inhibitors (the final concentrations are listed in the Results section). At the end of the exposure period the detached cells present in the culture media was removed and placed in a test tube. The adhered cells were detached using trypsin and were combined with the detached cells. The cells were centrifuged (200 g for 5 min at 20 °C) and washed with PBS to remove any excess media. The cells were then suspended in 100 μL incubation buffer (10 mM Hepes, 140 mM NaCl and 5 mM CaCl_2_, pH 7.4) containing 4 μL fluorescein isothiocyanate-labelled annexin V for 15 min at room temperature. Then 400 μL incubation buffer plus 10 μL PI (50 μg/mL) was added to the cells, which were filtered through gauze prior to being analysed on a Becton Dickinson FACScan, flow cytometer.

### 4.7. Western Blot Assay

The sorted cell subpopulations (viable, apoptotic and necrotic) from the treated cultures were collected and washed free of collecting media as described above. Cell lysates were prepared by resuspending 10^6^ cells/200 μL NETN (20 mM Tris pH 8.0, 1 mM EDTA, 100 mM NaCl, 0.5% NP-40) supplemented with 300 mM NaCl and 5 μg/mL leupeptin, pepstatin A and aprotinin and 0.5 mM PMSF for 15 min at 4 °C. The lysate supernatants were diluted 1:5 with 5X SDS sample buffer and boiled for 3 min. Samples containing 20 μg protein were loaded onto 10% SDS-PAGE gels, then blotted onto nitrocellulose filters (Amersham Pharmacia Biotech, Amersham, UK) using semi-dry electrotransfer. In order to resolve the cleaved bands of PARP, 5 μg lysates from the Jurkat apoptotic lysate set were run in each Western blot. After transfer the membranes were blocked in 5% blotto (0.1% Tween 20 in PBS containing 5% skim milk powder) for 2 h and then incubated in primary antibody overnight at 4 °C using either anti PARP antibody, anti-actin antibody or anti-GADPH antibody diluted in 1% blotto. The next day, the membranes were given three 10 min washes in PBS plus 0.1% Tween 20 buffer, before being incubated in either anti-mouse Ig-peroxidase conjugated (in case of anti-PARP Western) or anti-rabbit Ig-peroxidase conjugated (in case of anti-Actin and anti-GAPDH Westerns) at room temperature for 2 h. GAPDH was probed in these blots to show the levels of protein in each lane. The membranes were then given three 10 min washes in PBS plus 0.1% Tween 20 buffer, prior to the bands being visualised using ECL as per the manufacturer’s instructions.

### 4.8. Peptidase Activity

The P9 nonapeptide (YVAAAVVSH), which is cognate to the *N*-terminal cleavage site of preproTGFα, was labelled with ^125^I as previously described [29]. The rate of P9 hydrolysis by the sorted cell populations was assayed as follows: 20 μL of labelled P9 (1 μL ^125^I-P9 (10 pmoles) in 20 μL HBSS) was added to a 1.5 mL microfuge tube along with 50 μL of FACS sorted cell population (prewarmed to 37 °C for 15 min) for 15 min (P9 final concentration 140 nM). The aminopeptidase inhibitor bestatin [24] was added to the assay to a final concentration of 10 μM. At the end of the experiment 40 μL of the reaction mixture was spotted on a multichannelled TLC plate. The plates were dried and the peptide fragments separated by ascending chromatography [butanol:H_2_O:acetic acid, 100:30:10, pH 2.6], then imaged and analysed by phosphorimaging analysis as previously described [22].

### 4.9. Caspase 3 Activity Studies

HeLa cell cultures were grown to near confluency in 60 mm petri dishes prior to being exposed to different doses of UVB-irradiation as described above. The cells were then given fresh media (RPMI plus 5% FBS) and inhibitors (final concentrations listed in the Results section). After 20 h, the detached cells present in the culture media was removed and placed in a test tube. The adhered cells were detached using trypsin and were combined with the detached cells. The cells were centrifuged (200 for 5 min at 20 °C) and washed with prior to being lysed in 1 mL lysis buffer (10 mM Hepes, 0.1% CHAPS, 5 mM DTT, 2 mM EDTA and 1 mM PMSF, pH 7.25) by sonication. Following centrifugation (2 min at 8500 *g*), 10 μL of the cell supernatant along with 0.2 mM DEVD-pNA and caspase reaction buffer (100 mM Hepes, 10% sucrose, 5 mM DTT, 0.1% CHAPS, pH 7.25) to a final volume of 100 μL. Assays were incubated at 37 °C and the changes in absorbance levels at 415 nm were measured over 3 h. Specific activity was calculated as the amount of enzyme that hydrolyzes 1 nmol substrate/h per mg cell protein.

### 4.10. Calculations

The pmoles of P9-derived peptides separated by TLC were quantified using phosphorimage analysis. The rates of P9 hydrolysis were expressed as a function of cellular protein levels. The significance of differences due to apoptotic agents on the hydrolysis of P9 by HeLa cell subpopulations was determined using Student’s *t*-test, and Bonferroni’s adjustment of this analysis when necessitated by multiple comparisons.

## 5. Conclusions

UVB radiation causes an increase of CSP activity in HeLa cells that are undergoing apoptosis, while there was a loss of activity in those cells that underwent necrosis. The most active peptidase found on the cell membrane was an aminopeptidase. This increase in CSP activity was not due to activation of caspase 3, PARP cleavage, eversion of phosphatidylserine on the cell membrane, nor due to the cleavage of actin in the cell cytoskeleton. CSP activity was not activated in the apoptotic cells when the PARP inhibitor 3-aminobenzamide was added to the cultures, but was activated in the presence of the caspase 3 inhibitor (DEVD), though the mechanism(s) involved is/are unknown. Through further examination of the mechanism by which CSP activity is elevated in UV-irradiated skin cells undergoing apoptosis, it may be possible to devise inhibitors that can reduce the release of bioactive molecules from these cells, which may stimulate the growth of damaged cells, thereby in turn reducing the development on skin cancers.

## Figures and Tables

**Table 1:** Effect of different agents on the protein content of different HeLa cell subpopulations.

	Protein Content (mg/10^7^ cells) of Sorted Cell Subpopulations				
	
Agent	Viable (V)	Apoptotic (A)	Ratio (A/V)	Necrotic (N)	Ratio (V/N)
**0 J UVB-irradiated cells**					
Control	5.8 ± 0.1 (26)			2.1 ± 0.1 ^z^ (18)	0.36
Bestatin (10 μM)	6.0 ± 0.3 (14)			2.2 ± 0.1 ^z^ (8)	0.37
BB3103 (20 μM)	5.5 ± 0.2 (12)			2.1 ± 0.3 ^z^ (10)	0.38
3AB (10 mM)	5.8 ± 0.1 (16)			1.7 ± 0.1 ^z^ (10)	0.29
DEVD (1 μM)	5.5 ± 0.1 (20)			2.0 ± 0.2 ^z^ (10)	0.36
**500 J UVB-irradiated cells**					
Control	4.8 ± 0.2 (24)	4.0 ± 0.1 ^z^ (44)	0.83	1.9 ± 0.1 ^z^ (32)	0.40
Bestatin (10 μM)	4.5 ± 0.6 (8)	4.5 ± 0.2 (14)	1.00	1.8 ± 0.1 ^z^ (10)	0.40
BB3103 (20 μM)	4.2 ± 0.2 (10)	3.9 ± 0.1 (16)	0.93	2.2 ± 0.2 ^z^ (12)	0.52
3AB (10 mM)	4.8 ± 0.3 (16)	4.4 ± 0.1 ^z^ (20)	0.92	2.2 ± 0.1 ^a,z^(14)	0.46
DEVD (1 μM)	3.2 ± 0.2 ^a^ (12)	3.1 ± 0.1 ^a^ (20)	0.97	1.6 ± 0.2 ^z^ (10)	0.50

The statistical significance of the difference between the level of protein in the control population and the treated groups (between groups) are represented as ^a^ *p* < 0.0125 (calculated using Bonferroni’s adjustment for multiple comparisons with Student’s *t*-test), while the differences between the viable *vs*. the apoptotic or necrotic populations within treatment groups are represented as ^z^ *p* < 0.05.

**Table 2:** Effect of different treatments on cell surface peptidases (CSP) activity in HeLa cell subpopulations. Results represent the mean ± SD for 2–10 separate experiments, as seen in parantheses.

		P9-Derived Peptide Fragment (pmol/mg cell protein/15 min)			
		
Treatment	Cell Population	P1	P2	P3	P5
**0 μM Bestatin**					
**Control**	Viable (10)	139.0 ± 10.9	15.1 ± 1.2	5.1 ± 0.8	7.3 ± 1.2
	Apoptotic				
	Necrotic (7)	125.4 ± 25.3	26.3 ± 8.7	18.1 ± 3.3 ^z^	21.2 ± 4.6 ^z^
**Bestatin (10 μM)**	Viable (3)	113.8 ± 9.7 ^a^	29.2 ± 6.1 ^a^	3.7 ± 1.0	8.2 ± 0.7
	Apoptotic				
	Necrotic (2)	111.6 ± 62.6	21.7 ± 9.2	14.9 ± 8.0	14.5 ± 2.5
**BB3103 (20 μM)**	Viable (2)	104.4 ± 10.2 ^a^	10.2 ± 2.6	4.7 ± 1.5	10.8 ± 6.5
	Apoptotic				
	Necrotic (2)	82.6 ± 60.7	10.6 ± 8.2 ^a^	12.2 ± 5.0	17.6 ± 8.8
**3AB (1 mM)**	Viable (3)	106.9 ± 23.3	12.9 ± 4.9	5.8 ± 1.3	7.1 ± 0.7
	Apoptotic				
	Necrotic (2)	36.4 ± 24.8 ^a^	15.9 ± 3.0	8.0 ± 3.7	21.8 ± 16.4
**DEVD (1 μM)**	Viable (4)	172.4 ± 21.8 ^a^	14.3 ± 2.4	3.9 ± 1.3	6.8 ± 2.1
	Apoptotic				
	Necrotic (2)	259.0 ± 76.8 ^a^	33.3 ± 3.7 ^a^	15.2 ± 0.7 ^a^	16.0 ± 12.9
**10 μM Bestatin**					
**Control**	Viable (10)	14.5 ± 2.2	38.5 ± 3.3	50.8 ± 3.1	10.2 ± 1.5
	Apoptotic				
	Necrotic (7)	23.8 ± 3.3 ^z^	17.9 ± 3.2 ^z^	47.8 ± 9.1	22.1 ± 5.2 ^z^
**Bestatin (10 μM)**	Viable (3)	21.5 ± 4.1 ^a^	39.7 ± 9.1	58.2 ± 12.3	12.1 ± 1.6
	Apoptotic				
	Necrotic (2)	45.0 ± 26.6 ^a^	32.6 ± 19.2	62.2 ± 34.4	23.4 ± 7.5 ^a^
**BB3103 (20 μM)**	Viable (2)	33.4 ± 22.1	14.1 ± 2.2 ^a^	69.2 ± 36.6	18.1 ± 9.4
	Apoptotic				
	Necrotic (2)	31.1 ± 23.6	8.5 ± 6.4 ^a^	45.9 ± 31.6	18.4 ± 11.5
**3AB (1 mM)**	Viable (3)	13.9 ± 2.5	30.2 ± 1.8 ^a^	51.5 ± 7.5	12.6 ± 1.6
	Apoptotic				
	Necrotic (2)	21.3 ± 16.2	26.2 ± 18.8	40.8 ± 28.3	20.2 ± 16.0
**DEVD (1 μM)**	Viable (4)	9.5 ± 2.6 ^a^	42.3 ± 6.6	52.3 ± 6.5	6.4 ± 2.9 ^a^
	Apoptotic				
	Necrotic (2)	23.1 ± 20.3	67.4 ± 1.0 ^a^	151.0 ± 10.8 ^a^	24.2 ± 1.1 ^a^

The significance of differences between control *vs*. treated subpopulations are represented as ^a^ *p* < 0.05, while that between viable *vs*. necrotic subpopulations of the control groups are represented by ^z^ *p* < 0.05.

**Table 3:** Effect of different treatments on CSP activity in UVB-irradiated HeLa cell subpopulations. Results represent the mean ± SD for 2–16 separate experiments, as seen in parantheses.

		P9-Derived Peptide Fragment (pmol/mg cell protein/15 min)			
		
Treatment	Cell Population	P1	P2	P3	P5
**0 μM Bestatin**					
**Control**	Viable (8)	154.1 ± 14.9	21.1 ± 3.5	12.9 ± 5.1	9.9 ± 1.8
	Apoptotic (16)	275.9 ± 20.6 ^z^	28.8 ± 2.9 ^z^	8.1 ± 1.1	9.7 ± 1.5
	Necrotic (10)	6.7 ± 1.3 ^z^	5.2 ± 0.9 ^z^	5.1 ± 0.7 ^z^	7.6 ± 1.6
**Bestatin (10 μM)**	Viable (2)	92.8 ± 40.0 ^a^	14.3 ± 1.2 ^a^	10.7 ± 8.8	8.7 ± 5.6
	Apoptotic (4)	323.4 ± 105.7	59.2 ± 20.3 ^a^	9.0 ± 3.4	8.2 ± 1.2
	Necrotic (3)	7.2 ± 3.3	8.8 ± 3.1	9.5 ± 3.8 ^a^	1.8 ± 0.4 ^a^
**BB3103 (20 μM)**	Viable (2)	149.9 ± 5.4	5.9 ± 1.6 ^a^	6.5 ± 2.7	12.6 ± 8.3
	Apoptotic (4)	221.5 ± 22.4 ^a^	7.0 ± 1.0 ^a^	7.5 ± 3.1	15.3 ± 3.9 ^a^
	Necrotic (3)	5.4 ± 1.5	4.2 ± 1.6	4.0 ± 0.3	6.7 ± 1.7
**3AB (1 mM)**	Viable (4)	82.4 ± 6.3 ^a^	11.1 ± 0.7 ^a^	4.0 ± 0.7 ^a^	8.4 ± 0.9
	Apoptotic (4)	107.3 ± 10.7 ^a^	13.5 ± 2.5 ^a^	7.6 ± 0.3	11.2 ± 1.5
	Necrotic (3)	32.5 ± 5.8	4.2 ± 1.7	7.0 ± 2.9	7.6 ± 2.4
**DEVD (1 μM)**	Viable (2)	231.0 ± 41.4 ^a^	30.8 ± 3.1	22.2 ± 3.8	21.0 ± 5.5 ^a^
	Apoptotic (5)	350.7 ± 17.2 ^a^	40.0 ± 2.7 ^a^	10.5 ± 2.8	12.9 ± 2.6
	Necrotic (2)	8.1 ± 5.9	6.3 ± 3.5	7.2 ± 1.5	5.4 ± 1.5
**10 μM Bestatin**					
**Control**	Viable (8)	27.7 ± 7.5	29.5 ± 4.2	67.7 ± 9.7	15.4 ± 2.4
	Apoptotic (16)	33.5 ± 4.6	89.1 ± 10.8 ^z^	138.8 ± 12.5 ^z^	17.2 ± 1.8
	Necrotic (10)	3.3 ± 0.6 ^z^	4.1 ± 0.5 ^z^	6.8 ± 1.4 ^z^	4.5 ± 0.8 ^z^
**Bestatin (10 μM)**	Viable (2)	36.5 ± 19.3	20.4 ± 0.3 ^a^	50.5 ± 17.1	18.2 ± 8.4
	Apoptotic (4)	47.1 ± 11.1	131.8 ± 48.7	175.3 ± 55.7	15.3 ± 2.8
	Necrotic (3)	3.1 ± 1.5	3.9 ± 1.6	8.2 ± 3.8	4.7 ± 1.8
**BB3103 (20 μM)**	Viable (2)	39.9 ± 13.0	5.4 ± 0.9 ^a^	58.3 ± 10.7	24.9 ± 10.3
	Apoptotic (4)	72.5 ± 12.9 ^a^	9.9 ± 1.2 ^a^	119.4 ± 9.7 ^a^	34.1 ± 9.0 ^a^
	Necrotic (3)	3.8 ± 1.5	4.4 ± 1.8	7.7 ± 0.3	8.1 ± 3.0
**3AB (1 mM)**	Viable (4)	11.0 ± 1.5 ^a^	17.9 ± 2.0 ^a^	34.0 ± 3.6 ^a^	11.6 ± 1.7
	Apoptotic (4)	18.3 ± 0.9 ^a^	24.2 ± 5.8 ^a^	56.7 ± 5.7 ^a^	14.8 ± 2.4
	Necrotic (3)	8.4 ± 3.6	6.5 ± 0.7	14.5 ± 5.2 ^a^	9.6 ± 1.0 ^a^
**DEVD (1 μM)**	Viable (2)	58.9 ± 9.1 ^a^	58.0 ± 0.2 ^a^	129.4 ± 11.1 ^a^	27.4 ± 1.0 ^a^
	Apoptotic (5)	37.4 ± 12.0	150.0 ± 11.6 ^a^	204.7 ± 26.8 ^a^	15.7 ± 4.5
	Necrotic (2)	12.1 ± 6.5 ^a^	7.5 ± 4.3	21.8 ± 13.8 ^a^	12.9 ± 9.0

The significance of differences between control *vs*. treated subpopulations are represented as ^a^ *p* < 0.05, while that between viable *vs*. apoptotic or necrotic subpopulations of the control groups are represented by ^z^ *p* < 0.05.
